# Pan-cancer inference of intra-tumor heterogeneity reveals associations with different forms of genomic instability

**DOI:** 10.1371/journal.pgen.1007669

**Published:** 2018-09-13

**Authors:** Franck Raynaud, Marco Mina, Daniele Tavernari, Giovanni Ciriello

**Affiliations:** 1 Department of Computational Biology, University of Lausanne, Lausanne, Switzerland; 2 Swiss Institute of Bioinformatics, Lausanne, Switzerland; Hopp-Children’s Cancer Center KiTZ and German Cancer Research Center DKFZ, GERMANY

## Abstract

Genomic instability is a major driver of intra-tumor heterogeneity. However, unstable genomes often exhibit different molecular and clinical phenotypes, which are associated with distinct mutational processes. Here, we algorithmically inferred the clonal phylogenies of ~6,000 human tumors from 32 tumor types to explore how intra-tumor heterogeneity depends on different implementations of genomic instability. We found that extremely unstable tumors associated with DNA repair deficiencies or high chromosomal instability are not the most intrinsically heterogeneous. Conversely, intra-tumor heterogeneity is greatest in tumors exhibiting relatively high numbers of both mutations and copy number alterations, a feature often observed in cancers associated with exogenous mutagens. Independently of the type of instability, tumors with high number of clones invariably evolved through branching phylogenies that could be stratified based on the extent of clonal (early) and subclonal (late) instability. Interestingly, tumors with high number of subclonal mutations frequently exhibited chromosomal instability, *TP53* mutations, and APOBEC-related mutational signatures. Vice versa, mutations of chromatin remodeling genes often characterized tumors with few subclonal but multiple clonal mutations. Understanding how intra-tumor heterogeneity depends on genomic instability is critical to identify markers predictive of the tumor complexity and envision therapeutic strategies able to exploit this association.

## Author summary

Cancer is characterized by cells accumulating molecular alterations promoting specific phenotypic features, such as uncontrolled proliferation and survival. Cancer cells sometimes exhibit a high number of such alterations, often driven by defects of the DNA repair pathway or by external mutagens, such as tobacco smoking or UV-radiation. Highly altered cells are termed genomically unstable. A major consequence of genomic instability is that a single tumor is often composed by cells that have accumulated distinct alterations. This diversity is termed intra-tumor heterogeneity and represents a critical clinical challenge. In this study, we examined how different forms of genomic instability are associated with intra-tumor heterogeneity. We inferred intra-tumor heterogeneity in ~6000 human tumors and found that tumors with extreme mutational or chromosomal instability were not the tumors with the highest number of clones. Instead, tumors harboring both mutational and chromosomal alterations were the most diverse. Furthermore, we identified specific genetic fingerprints that are associated with early and/or late genomic instability. These results show that cancer genomic instability does not necessarily lead to high intra-tumor heterogeneity and, importantly, they provide markers to recognize when it does.

## Introduction

Cancer is a dynamic and ever-changing disease that mutates and evolves during its progression [1]. While the transformation from healthy to malignant cell is characterized by a few selected oncogenic alterations [2], genomic instability is frequently observed in formed tumors, where it fuels the acquisition of novel molecular changes diversifying the cancer cell population [3]. As a result, each tumor is a composite of multiple *clones*, defined as groups of cells that are genetically identical within each group, but different among them [4].

Genomic instability has been long considered a major driver of intra-tumor heterogeneity. Multiple implementations of genomic instability have been identified and characterized in tumors [5]. These differ by type of genetic lesions being accumulated, e.g. somatic mutations [6] or copy number alterations [7], as well as by the extent of time and space throughout the genome that is affected by these lesions [8], [9]. Importantly, recent studies have reported diverse association between specific types of genomic instability and clinical outcome. In particular, chromosomal instability was found indicative of worse prognosis in lung adenocarcinoma and other diseases [10], [11], even though tumors with extreme mutational or chromosomal instability were reported having better prognosis than less altered tumors in multiple tumor types [6], [12–14]. Genomic instability therefore encompasses diverse molecular phenotypes associated with distinct mutational processes and clinical outcome. Whether these phenotypes are associated with diverse extent and patterns of intra-tumor heterogeneity remains an outstanding question.

Approaches based on single-cell profiles [15–18] or multiple biopsies of the same tumor [19–21] have revealed a daunting diversity among cancer cells. Unfortunately, single-cell analyses of tumors or profiling of multiple samples for each patient face technical and cost limitations, thus large scale datasets of these types are currently limited for systematic investigations. In response to these limitations, algorithmic approaches have been proposed to infer the clonal composition of a tumor from the genetic profile of a single sample [22–26]. Using such tools, different clonality and timing of emergence have been shown for specific therapeutically actionable mutations [27] and an association has been found between intra-tumor heterogeneity and patients’ prognosis [28].

Here, we used computational inference of intra-tumor heterogeneity to explore its association with genomic instability. Briefly, we collected data for 5,593 human cancer genomes from 32 tumor types profiled by The Cancer Genome Atlas (TCGA) (S1 Table) and inferred the clonal composition of each tumor from its repertoire of somatic mutations and copy number alterations. The resulting cohort of tumor clonal phylogenies allowed us to assess how intra-tumor heterogeneity depends on diverse forms of genomic instability and whether these are associated to specific genetic lesions or mutational signatures that can act as markers of the underlying tumor complexity.

## Results

To estimate intra-tumor heterogeneity in individual tumor samples from their repertoire of somatic mutations and copy number changes, we used a combination of two algorithmic approaches. First, we used ABSOLUTE [29] to integrate mutations and copy number changes of each tumor and determine copy number statuses of mutated and wild-type alleles. Then, we used PhyloWGS [24] to infer the clonal architecture of a tumor from its set of mutations and copy number alterations. Briefly, PhyloWGS defines clones as groups of cells sharing mutations with identical or similar variant allele frequencies (VAF) after accounting for the copy number statuses of variant and wild-type alleles. Notably, this approach was validated on real and simulated tumors exhibiting variable numbers of mutations and read-depths, including cases in the range of the exome sequencing data used in this study [24]. To increase the robustness of our results, we estimated the clonal structure of each TCGA tumor sample based on the set of top scoring predictions made by multiple runs of PhyloWGS, each weighted by its likelihood (see Methods).

**Fig 1:**
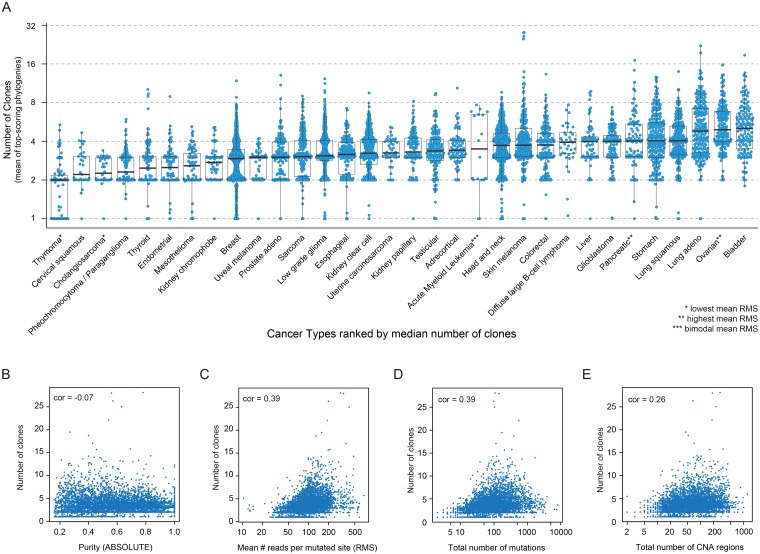
Number of clones in human tumors. **A)** Number of clones in human tumors within each tumor type. Tumor types are ranked by median number of clones. The number of clones in each human tumor is the weighted mean of the number of clones obtained in the top scoring PhyloWGS phylogenies for that sample. The thick central line of each box plot represents the median number of significant motifs, the bounding box corresponds to the 25th–75th percentiles, and the whiskers extend up to 1.5 times the interquartile range. **B-E)** Correlation between number of inferred clones by PhyloWGS (Y-axis) and tumor purity (**B**) inferred by ABSOLUTE, mean number of reads per mutated sites (RMS) (**C**), number of mutations (**D**) and number of copy number altered segments (**E**) (X-axes).

Within the TCGA dataset, the inferred number of clones ranged between 1 and 28, with 95% of the cases having less than 8 clones (Fig 1A and S1 Table). Both the range of number of clones and the ranking of tumor types by mean number of clones was in high agreement with a previous study where a different algorithmic approach was used to infer intra-tumor heterogeneity on a smaller dataset [28]. Furthermore, molecular tumor subtypes often exhibited distinct intra-tumor heterogeneity (S1A Fig).

Both tumor purity and sequencing depth have been shown to affect the inference of tumor clonality. In our dataset, tumor purity estimated by ABSOLUTE did not correlate with the estimated number of clones (Spearman’s correlation coefficient, cor_S_ = -0.07, Fig 1B) indicating that the combination of approaches here used are robust to variable tumor content. Conversely, estimated number of clones per tumor were positively correlated with the mean number of reads covering a mutated site (mean RMS) (cor_S_ = 0.39, Fig 1C) confirming that deep sequencing is beneficial to detect rare variants and clones.

To assess the impact of variable mean RMS on our results, we first compared mean RMS values among tumor types (S1B Fig). Cancer types with the lowest (cholangiosarcoma and thymoma) and highest (ovarian and pancreatic cancer) mean RMS scored respectively among the least and most heterogeneous (Fig 1A), suggesting their ranks could here depend on the sequencing coverage. However, the overall ranking of tumor types by mean number of clones was not strongly associated with the ranking by mean RMS (S1B Fig) and mean RMS values were similar among the majority of tumor types. Next, we explored mean RMS within each tumor type. The majority of tumor type exhibited only a moderate correlation between mean RMS and inferred number of clones (60% had cor_S_ < 0.4, 85% had cor_S_ < 0.5), with the notable exception of acute myeloid leukemia (AML, cor_S_ = 0.75). Indeed, in AML, a subset of samples exhibited high clonal heterogeneity (>4 clones), a 2.5-fold increase of RMS, and a 15-fold increase in number of mutations. Overall, these results suggest that caution should be taken when comparing inferred intra-tumor heterogeneity among tumors with heterogeneous sequencing coverage. Nonetheless, with the exception of a few tumor types, mean RMS were rather similar among and within the tumor types that we analyzed and did not have a major impact on mutation calling (correlation between mean RMS and number of mutation, cor_S_ = −0.07) or on inferred clonal heterogeneity.

The rank of tumor types based on their mean number of clones (Fig 1A) bore a striking resemblance with a previously reported rank based on mutation load [30], indicating that most mutagenic tumor types are on average also the most clonally diverse. Indeed, the inferred number of clones correlated with the total number of mutations per sample (corS = 0.39, Fig 1D), even though the most frequently mutated tumor samples were not the most clonally diverse. In addition, we found a positive correlation between the number of copy number alterations and number of clones (corS = 0.26, Fig 1E). These results indicate that intra-tumor heterogeneity can be driven by both mutational and chromosomal instability.

**Fig 2:**
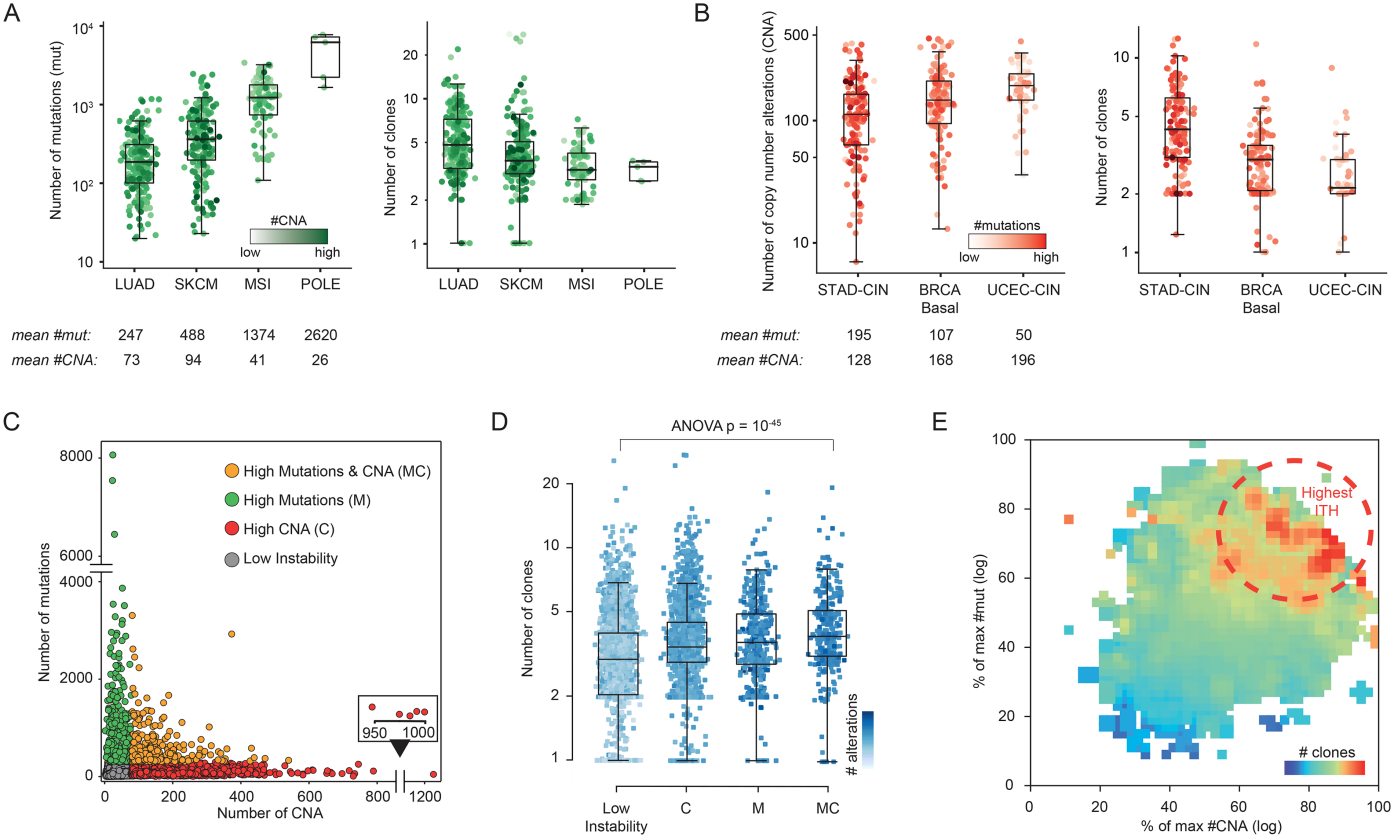
Genomic instability and intra-tumor heterogeneity. **A)** Number of mutations (left panel) and number of clones (right panel) in tumor types and subtypes with high mutation instability. Samples are color coded by the their number of copy number changes, with high color intensity corresponding to high number of events. **B)** Number of copy number altered segments (left panel) and number of clones (right panel) in tumor types and subtypes with high chromosomal instability. Samples are color coded by the their number of mutations, with high color intensity corresponding to high number of events. **C)** Total number of mutations (Y-axis) versus of copy number altered segments (CNA, X-axis) for all tumor samples (n = 5593). Samples are grouped 4 classes: low numbers of mutations (<300) and CNA (<80) (gray), high number of mutations (>300) and low number of CNA (<80) (M class, green), high number of CNA (>80) and low number of mutations (<300) (C class, red), or high numbers of both mutations (>300) and CNA (>80) (MC class, orange). **D)** Number of clones in classes M, C, MC, and with Low Instability. Samples are color coded by the their total number of alterations, with high color intensity corresponding to high number of events. **E)** The mean number of clones increases (from cold to warm colors) in samples with relatively high numbers of both mutations and CNA. Axes are normalized by the maximum of the logarithm of the number of mutations (Y-axis) and CNA (X-axis). **Acronyms**: LUAD: lung adenocarcinoma, SKCM: skin melanoma, MSI: microsatellite instability, POLE: tumors with hotspot mutations of polymerase-ε gene, STAD: stomach adenocarcinoma, BRCA: breast cancer, UCEC: endometrial cancer, CIN: chromosomal instability.

To correlate the inferred number of clones with different forms of genomic instability, we explored distinct highly mutagenic and chromosomally unstable tumor subtypes. High mutation loads have been associated with exogenous carcinogens, such as tobacco smoking in lung adenocarcinoma (LUAD) and UV radiation in skin melanoma (SKCM), as well as with defects of the DNA repair machinery, such as mismatch repair deficiency in micro-satellite unstable tumors (MSI) or specific mutations affecting the polymerase-ε encoding gene *POLE*. MSI and especially *POLE*-mutant tumors in our dataset exhibited over one order of magnitude more mutations than lung or melanoma tumors (Fig 2A—left panel), however they were characterized by fewer clones (Fig 2A—right panel). Similarly, when we compared stomach (STAD), breast (BRCA), and serous endometrial (UCEC) tumors exhibiting high chromosomal instability (CIN), we found that the subgroup with the least amount of copy number changes (STAD) was the one inferred having the greatest number of clones (Fig 2B).

We previously reported that at the extreme of genomic instability tumors exhibit high number of mutations (e.g. MSI and *POLE*-mutant cases) or high numbers of copy number alterations (e.g. serous endometrial cancer), but never both [31]. In our dataset, we could confirm an inverse trend between the accumulation of copy number changes and somatic mutations (Fig 2C). Interestingly, upon partitioning all samples based on the number and type of genetic alterations (Fig 2C), we found that lung, melanoma, and CIN stomach cancer were over-represented in the class exhibiting a relatively high number of both mutations and copy number changes (S2 Fig) and indeed these tumor types tend to exhibit high number of both type of alterations, even though not as extreme as in other subtypes (Fig 2A and 2B).

Consistent with what we observed for these specific tumor subtypes, we found that tumors exhibiting high numbers, yet not extreme, of both mutations and copy number alterations were predicted being the most intrinsically heterogeneous (Fig 2D) and this trend was confirmed by an independent approach in a subset of our cohort (S3 Fig) [28]. Independently of the cut-offs used to partition our tumor dataset, we confirmed that the highest inferred numbers of clones were in tumors where both numerous mutations and copy number alterations were concurrently observed (Fig 2E).

### From clone sets to clonal architectures

**Fig 3:**
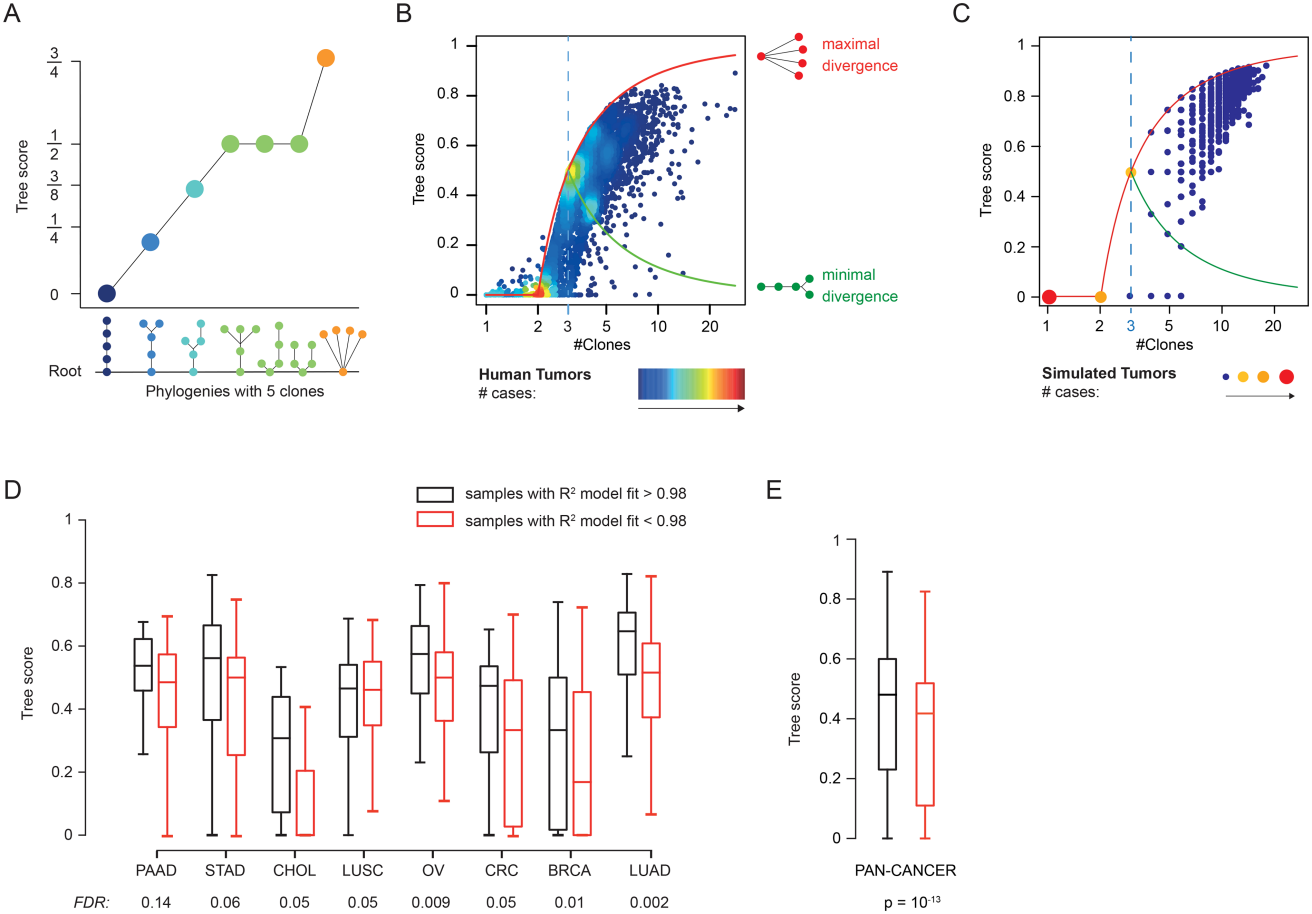
Linear and divergent evolution for low and high number of clones. **A)** Example of Tree score values for phylogenies with 5 clones. The Tree scores increase with increasing divergence. **B-C)** Tree score as a function of the number of clones observed in human (**B**) and simulated (**C**) tumors. Divergent phylogenies can emerge when at least 3 clones are detected (blue dotted line). The range of Tree scores for phylogenies with more than 3 clones goes from a minimal divergence value (green line) to a maximal divergence value (red line). Points are colored to reflect point density with cold colors for low density and warm colors for high density. **D)** Box plot comparison of Tree scores in samples exhibiting features of neutral evolution (R^2^ model fit > 0.98, black) and samples that do not exhibit such features (R^2^ model fit < 0.98, red) in individual cancer types that where the difference was significant (FDR < 0.2, left) and across the whole dataset (right). **Acronyms**: PAAD: pancreatic adenocarcinoma, LUAD: lung adenocarcinoma, STAD: stomach adenocarcinoma, BRCA: breast cancer, CHOL: cholangiosarcoma, LUSC: lung squamous-cell cancer, OV: ovarian cancer, CRC: colorectal cancer.

To further characterize how intra-tumor heterogeneity emerged in our set of tumors, we explored their inferred phylogenies, i.e. the ensemble of clone-to-clone relationships that describe which clone descends from the others. *Linear* phylogenies are the result of the sequential generation of clones along the same lineage, i.e. the last clone is the product and summary of all its predecessors. Conversely, in *branching* phylogenies multiple clones spur from the same common ancestor, generating independent lineages that can evolve in distinct populations with little similarity from one another. Tumor phylogenies are typically combinations of linear and branching evolution and they can be represented as connected graphs or *trees* where clones are the nodes of the tree and two clones are connected if one descends from the other. According to this representation, the first emerged clone is the *root* of the tree, while clones emerging last without descendants are the *leaves*. Intuitively, the more branching a phylogeny, the closer each leaf will be to the root, conversely perfectly linear phylogenies will have only one leaf at the maximal possible distance from its root. We formalized this intuition and quantify each phylogeny with the following score:

$$Tree\;score=1-\;\frac{\frac{1}{L}\sum_{l}d(l,root)}{N-1}$$

where *L* is the total number of leaves, *N* the total number of clones, and *d(l*, *root)* is the length of the path connecting a leaf *l* to the *root* of the tree. Based on this definition, all linear phylogenies will obtain a score equal to 0, while the Tree score will increase with its degree of branching and number of branching nodes (Fig 3A).

Tree scores computed for our tumor cohort indicated that branching phylogenies were almost invariably observed as the number of clones increased. In tumor predicted having more than 5 clones, linear phylogenies or phylogenies with minimal branching (i.e. linear phylogenies with only two branching leaves) were almost never observed (Fig 3B). Importantly, this association was independent of the type of genomic instability (S4A Fig). Notably, as for the number of clones, genomically unstable tumor subtypes exhibiting high numbers of both copy number alterations and mutations were associated with higher Tree score than tumors with extreme numbers of exclusively one type of alteration (S4B Fig). Within each tumor type, patients with Tree scores above the average did not show significantly different survival, except in 4 tumor types were a consistent trend was observed. Indeed, in all 4 cancer types, patients with high Tree scores exhibited on average 4 or more clones and were associated with better prognosis (S5A–S5D Fig), consistent with previous observations made on a subset of the TCGA cohort [28]. A stratification of patients based on low (<0.3) and high (>0.6) Tree scores confirmed that patients with high Tree scores had higher median overall survival than patients with low Tree score in the majority of the tumor types (S5E Fig).

Branching phylogenies have been previously reported to be associated with the clonal expansion that characterizes tumor progression, rather than initiation [32]. To verify this association, we used a previously proposed mathematical model of tumor progression [33] based on two parameters: the mutation rate μ and fitness *s* (see Methods). Briefly, at each iteration cells can either replicate or die with complementary probabilities that depend on the number of driver mutations *k* and the fitness parameter *s* (the higher *k* and *s*, the higher the probability of replicating). Replicating cells will acquire a mutation with probability μ, and such mutation will be considered a driver with probability Kμ (here K = 0.025). Using this model, we simulated and characterized the evolution of approximately 40,000 simulated tumors spanning a wide range of evolutionary parameters (μ and *s*) (see Methods). Observed number of clones and Tree scores of the simulated tumors were remarkably concordant with the inferred values in the human cohort (Fig 3C), confirming that high intra-tumor heterogeneity emerging during exponential growth gives rise to branching phylogenies.

The model that we adopted allows the emergence of mutations improving the cell fitness (i.e. *driver*) and, thus, it mimics tumor evolution under selection. However, it has been proposed that a fraction of human tumors displays features that can be explained exclusively by neutral evolution [34]. In our dataset, we detected samples with such features across all tumor types (S5F Fig). Interestingly, in 8 distinct tumor types we found that tumors exhibiting features of neutral evolution had significantly higher Tree scores than tumors without such features (Fig 3D), whereas the opposite association was never observed. This trend was confirmed in the pan-cancer cohort (Fig 3E) and suggests that neutral evolution could foster intra-tumor heterogeneity and the emergence of branching lineages.

### Clonal and subclonal genomic instability

Tumor phylogenies allow to explore the temporal emergence of individual or groups of mutations. In particular, previous characterizations of tumor phylogenies have focused on the dichotomy between clonal and subclonal mutations [10], [27], [35]. Clonal mutations are present in all cancer cells and are typically considered early events. Subclonal mutations emerge later during tumor evolution and thus characterize only individual or subsets of clones. Starting from this premise, we quantified the number of clonal and subclonal mutations for each tumor in our dataset, and explored whether different types of genomic instability are themselves early or late emerging events.

In our tumor phylogenies, clonal mutations are grouped in the root, which corresponds to the oldest detectable clone and it documents, at least in part, the previous history of the tumor. The root could either represent the first clone that underwent clonal expansion or the last one able to outcompete all previous clones, typically in association with an evolutionary bottleneck where cells undergo strong selective pressure (e.g. therapeutic intervention or metastatic migration).

**Fig 4:**
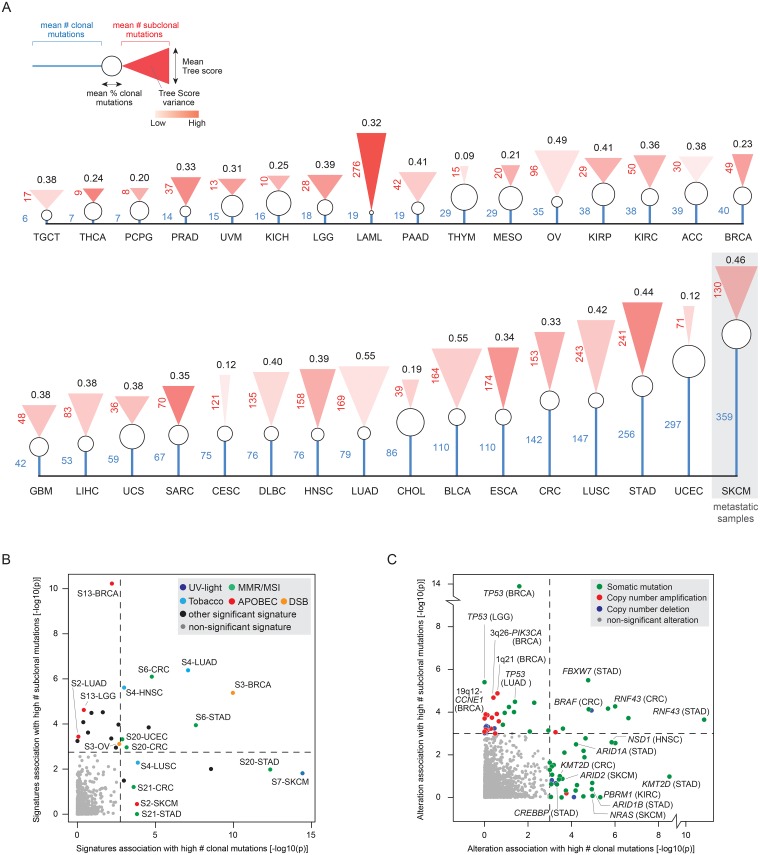
Clonal and subclonal genomic instability. **A)** Mean clonal and subclonal mutations found in each tumor type. For each tumor type we report: mean number of clonal mutations (blue line), mean number of subclonal mutations (height of the red triangle), mean Tree score (base width of the red triangle), and mean Tree score variance (shade of red within the triangle: intense red corresponds to high variance, transparent red corresponds to low variance). **B)** Statistical significance of the difference between the numbers of clonal (X-axis) and subclonal (Y-axis) mutations in patient exhibiting a specific mutational signature (S, n = 22) in each tumor type. Signatures of known etiology that scored as significant (p-value < 0.003, FDR < 0.1) in at least 2 tumor types are labeled and color coded based on their etiology, all other significant signatures are in black. Signatures below the significance threshold are in gray. **C)** Statistical significance of the difference between the numbers of clonal (X-axis) and subclonal (Y-axis) mutations in patient exhibiting a specific alteration (n = 505) in each tumor type. Significant alterations (p-value < 0.001, FDR < 0.25) are color coded based on their type as described in the legend. Alterations below the significance threshold are in gray. **Acronyms**: TGCT: testicular cancer, THCA: thyroid cancer, PCPG: pheochromocytoma / paraganglioma, PRAD: prostate cancer, UVM: uveal melanoma, KICH: kidney chromophobe cancer, LGG: low grade glioma, AML: acute myeloid leukemia, PAAD: pancreatic adenocarcinoma, THYM: thymoma, MESO: mesothelioma, OV: ovarian cancer, KIRP: kidney papillary cancer, ACC: adrenocortical cancer, BRCA: breast cancer, GBM: glioblastoma, LIHC: liver cancer, UCS: uterine carcinosarcoma, SARC: sarcoma, CESC: cervical cancer, DLBC: diffuse large B-cell lymphoma, HNSC: head and neck squamous-cell cancer, LUAD: lung adenocarcinoma, CHOL: cholangiosarcoma, BLCA: bladder cancer, ESCA: esophageal cancer, CRC: colorectal cancer, LUSC: lung squamous-cell cancer, STAD: stomach adenocarcinoma, UCEC: endometrial cancer, SKCM: skin melanoma.

Tumor types in our human dataset exhibited a variable average number of clonal mutations, with most of them ranging between 30 to 60% of their total number of mutations (Fig 4A and S1 Table). Cancers that exhibited the highest extent of intra-tumor heterogeneity, such as lung, bladder, and stomach cancers, were characterized by high numbers of both clonal and subclonal alterations indicating that genomic instability is here emerging early and continues to evolve as the tumor progresses. An interesting exception was skin melanoma which was characterized by the highest number of clonal mutations, consistent with all of these samples being metastatic and not primary tumors (Fig 4A). In this case, the root of the tumor phylogeny is likely to represent the clone that was able to migrate from an advanced primary tumor and seed the metastasis.

Next, we explored whether the emergence and selection of genomic alterations were associated with the extent of clonal and subclonal mutations. First, we estimated within each patients which mutational processes (or mutational signatures) could explain the emergence of the observed patterns of mutations [36], [37]. For each tumor type, we compared the numbers of clonal and subclonal mutations in patients exhibiting a given signature and in patients that did not (Fig 4B, S2 Table). As expected, the UV light signature (S7) was strongly associated with melanoma patients with high number of clonal mutations, consistent with these been mostly metastatic samples and characterized by high numbers of clonal events. Signatures characteristic of tumor subtypes with a high mutation load were associated with high numbers of both clonal and subclonal mutations. For example, lung and head neck cancer patients exhibiting a signature associated with tobacco smoking (S4) had higher number of both clonal and subclonal mutations than patients with smoke unrelated tumors, even though in lung squamous-cell cancer only the different numbers of clonal mutations reached statistical significance (FDR < 0.1). Similarly, DNA repair deficiencies, such as double strand break repair (DSB, S3) in breast and ovarian cancer, or mismatch repair (MMR, S6 and S20), in colorectal and stomach cancer were associated with higher number of both clonal and subclonal mutations. Interestingly, colorectal and stomach cancer patients exhibiting a signature of unknown etiology (S21) but associated with microsatellite instability (MSI) had significantly higher numbers of clonal, but not subclonal, mutations than patients without such signature. Finally, patients exhibiting signatures of APOBEC-associated mutagenesis (S13 and S2) had higher numbers of subclonal mutations in diverse tumor types, except for metastatic melanoma, consistent with this mutational process occurring late in tumor development [27].

Then, we performed a similar analysis to test whether the selection of ~500 cancer-associated mutations and copy number alterations [38] was associated with a high number of clonal or subclonal mutations (S2 Table). Surprisingly, alterations that were associated with a higher number of subclonal, but not clonal, events, included for the most part copy number changes (67%), especially in sarcomas, breast and ovarian cancers, and *TP53* mutations, in lung adenocarcinoma, low grade glioma, and breast cancer (Fig 4C). Alterations associated with high number of clonal, but not subclonal, events were instead prevalently recurrent mutations (87%), mostly occurring in colorectal and stomach cancer, and skin melanoma. Interestingly, these mutations were enriched for events targeting chromatin remodeling factors such as SWI/SNF components *PBRM1*, *ARID2*, *ARID1A*, and *ARID1B*, lysine methyltransferase KMT2D, and histone acetyltransferase *CREBBP* (Fig 4C). Finally, highly recurrent mutations in MSI tumors, such as those affecting *RNF43* and *BRAF* in gastric cancers [39], [40] were associated with high number of clonal and subclonal mutations, consistent with MSI tumors having a higher mutational load than micro-satellite stable tumors.

Overall, mutational signatures and cancer-associated alterations further highlighted that distinct patterns of genomic instability are associated with different extents of intra-tumor heterogeneity.

## Discussion

Intra-tumor heterogeneity is intrinsically difficult to measure as a limited portion of a tumor is typically accessible for molecular analyses, providing only a static snapshot of a disease in constant evolution. Computational techniques can help to infer tumor progression, extract shared evolutionary patterns through the analysis and comparison of large-scale sample cohorts, and predict the missing pieces of an otherwise incomplete picture. Nonetheless, these approaches often have limited power, especially if relying only on whole-exome sequencing of single samples, they depend on sequencing coverage and mutation calling, and still mostly rely on genetic data to infer clonal diversity. Based on a simple comparison of different tools that were applied to the same tumors, we observed that results on individual cases are often inconsistent, however, trends derived from the whole set of samples were reproducible.

In this study, we combined two different methods that used both mutation and copy number data to explore the association between intra-tumor heterogeneity and diverse forms of genomic instability. Surprisingly, tumors with the highest alteration burden were not found to be the most heterogeneous. Indeed, both mutational instability associated with DNA repair deficiencies and high chromosomal instability (CIN) were associated with less intra-tumor heterogeneity than instability associated with exogenous mutagens (e.g. tobacco smoke and UV-radiation). In particular, the most heterogeneous tumors were those concurrently exhibiting high, yet not extreme, numbers of mutations and copy number alterations. This molecular phenotype was common in lung and skin melanoma, but also bladder, head and neck and CIN stomach cancer (S2 Fig), and could represent a marker of high intra-tumor heterogeneity.

Tumors likely undergo multiple phases of clonal expansions and diversification punctuated by evolutionary bottlenecks (e.g. therapeutic intervention or nutrients depletion) where only one or a few clones harbor the necessary molecular features to survive. Computationally inferred phylogenies from single samples are thus likely representative of one such phase, but cannot capture the whole evolutionary history of the disease. This was nicely evidenced by the analysis of metastatic skin melanoma, where the large numbers of clonal mutations likely resulted from the development and progression of heterogeneous primary tumors, out of which a clone was able to seed the metastasis. Within this context, the distinction between clonal and subclonal mutations, provided us with a simple but useful means to explore the early versus late emergence of genomic instability. However, tumor phylogenies were here inferred from a single sample from each tumor, hence mutations that appeared as clonal, might actually be only “locally clonal”, i.e. different regions of the same tumor might not exhibit such mutations or exhibit them only in a fraction of cells.

Overall, tumors with greatest intra-tumor heterogeneity exhibited high numbers of both clonal and subclonal mutations, suggesting that genomic instability emerged early, but was sustained and fostered during tumor evolution. An interesting case was represented by gastric tumors with microsatellite instability (MSI). MSI tumors are associated with mismatch repair deficiency, which has been associated with multiple signatures (S6, S20, S15, S21, and S26) [41]. Nonetheless, the extent of clonal and subclonal mutations associated with these signatures were different, especially between signatures S6 and S21 (Fig 4B), potentially suggesting the existence of distinct MSI subtypes associated with different mutational processes. On the other hand, we found that chromosomal instability characterized by multiple copy number changes and *TP53* mutations, was often accompanied by multiple subclonal mutations confirming previous observations in glioma [42] and extending them to other tumor types. Moreover, amplification of TP53 inhibitory proteins MDM2 (12q15) and MDM4 (1q32) and deletion of the MDM2 inhibitor ARF (*CDKN2A*, 9p21) exhibited a trend for being associated with high numbers of subclonal mutations in 8 tumor types (p value < 0.1, S2 Table). Interestingly, while *TP53* mutations or alterations in the p53 pathway are invariably observed in chromosomally unstable tumors [31], only few other mutations have been reported as recurrent in these tumor subtypes, suggesting these multiple subclonal events might be only a “passenger” byproduct of p53 deficiency.

Targeted sequencing of cancer-associated variants is empowering clinicians with the ability to tailor therapeutic protocols to the genetic fingerprint of each tumor. These decisions however often rely on a single and potentially incomplete observation. While single-cell sequencing or multiple sampling of the same tumor are still for the most part unfeasible in the clinic, the identification of tumors at “high-risk” of intra-tumor heterogeneity could provide a means to better prioritize patients likely to benefit from additional analysis and profiling. Genomic instability has been often proposed as a major driver of intra-tumor heterogeneity and, thus, as a potential marker of its extent. Our study delved into the diverse implementations of such instability and characterized their potential to anticipate low or high intra-tumor heterogeneity. With a more comprehensive understanding of the risks and vulnerabilities posed by highly unstable genomes, strategies can be envisioned to exploit these phenotypes to control intra-tumor heterogeneity and enhance therapeutic response.

## Materials and methods

### TCGA cohort

Molecular data for the tumor types analyzed in this study has been collected from the FireHose (https://gdac.broadinstitute.org/) and cBioPortal (Cerami et al., 2012) (http://www.cbioportal.org/) data repositories for The Cancer Genome Atlas (TCGA). Mutation files (MAF format) and copy number segmentation files used for the analyses in this manuscript are available at https://zenodo.org/record/1404658#.W4VNVJMzbOQ. Reported numbers of mutations per sample (S1 Table) include all variants, reported numbers of copy number alterations correspond to the number of segment with copy number value > 0.3 (gain) or < -0.3 (loss).

### Software

#### Inference of tumor phylogenies: PhyloWGS [24], numerical procedure and scoring

PhyloWGS is a method to infer evolutionary relationships between clonal subpopulations based on variant allele frequencies of point mutations and taking into account copy number alterations at the mutated loci. PhyloWGS provides in output detailed phylogenies representing the clonal evolution, thus inferring the clonal architecture and not only the clonal composition of each tumor. In particular, PhyloWGS does not provide a unique tree representing the phylogenetic evolution of the tumor, but a number of trees, each scored by its complete-data log likelihood. For each sample, we run 10 inference procedures with different seeds and we kept the 50 trees with the highest complete-data log likelihood for each run for a total of 500 phylogenies for each human tumor. We then sorted all the trees by log-likelihood and kept the top 10% (50 trees) for further analysis. For the reduced list of trees, we assigned a score $S_{50}^{i}$ to each tree i according to:

$$S_{50}^{i}=\frac{{CDLL}_{50}^{i}-min({CDLL}_{50})}{\text{max}\left({CDLL}_{50}\right)-\text{min}\left({CDLL}_{50}\right)}$$

where CDLL^i^_50_ is the complete-data log likelihood of the tree i and min(CDLL_50_) (resp. max(CDLL_50_)) is the minimum (resp. maximum) complete-data log likelihood value within the reduced set of trees. For each sample, we computed the weighted average number of clones and weighted average Tree score as follows:

$$\#Clones=\frac{1}{\sum_{i=1}^{50}S_{50}^{i}}\;\sum_{i=1}^{50}S_{50}^{i}C^{i}$$

$$Tree\;score=\frac{1}{\sum_{i=1}^{50}S_{50}^{i}}\;\sum_{i=1}^{50}S_{50}^{i}T^{i}$$

where C^i^ is the number of clones and T^i^ the Tree score for the tree *i* as defined in the main text.

#### Accuracy of PhyloWGS

PhyloWGS accuracy depends on both the number of mutations and the sequencing read depth. In the original publication, PhyloWGS was applied to synthetic data with known clonal structures to test whether the method was able to recover the true number of clones based on the number of mutations and the read depth. Based on their results, we extract threshold lines for different number of clones in the population separating regions where the reconstruction is accurate and where it is not (S6 Fig). For tumors falling above the threshold line, the reconstruction is considered accurate, whereas below the threshold line the number of clones is likely to be overestimated. The vast majority of the TCGA samples we analyzed are in the region of accurate phylogenetic reconstruction. A few cases with high heterogeneity (number of clones > 6) fall slightly below the threshold line indicating a potential overestimation of one clone. While PhyloWGS was designed for whole genome data, the authors demonstrated that it did not necessary require thousands of mutations from whole genome but instead could provide accurate reconstruction for number of mutations and read depth similar to those from TCGA dataset. We used PhyloWGS with the default parameters.

#### Absolute

We used ABSOLUTE [29] to estimate the copy number status of each point mutation. Originally, ABSOLUTE was designed to infer purity and ploidy of tumor samples, but it also returns information on the copy number status of point mutations when a list of mutations is provided as input. ABSOLUTE reports multiple possible solutions and often manual curation is required to select the best among the top ones (personal communication). For this reason, in this study we relied on TCGA samples with purity and ploidy values previously reported by the authors of the original publication. We independently ran ABSOLUTE on all samples and for each sample i selected the solution that minimizes:

$${\left({Pur}_{i}^{abs}-\;{Pur}_{i}^{TCGA}\right)}^{2}+\;{\left({Plo}_{i}^{abs}-\;{Plo}_{i}^{TCGA}\right)}^{2}$$

where (Pur^abs^_i_, Plo^abs^_i_) is the purity and the ploidy obtained from our ABSOLUTE runs and (Pur^TCGA^_i_, Plo^TCGA^_i_) is the purity and the ploidy previously reported for the sample i.

### Modeling cancer evolution

To model cancer evolution, we rely on the model proposed by Bozic et al. [33]. This model is a discrete time Galton-Watson branching process in which cells can at each time step either replicate (with a probability b) or die (with a probability d). During the replication, one of the two daughter cells can acquire a new alteration with a probability μ. If an alteration occurs, this can be of two types: a driver alteration confers to the cell a selective advantage by reducing its probability to die, while a passenger mutation has a neutral effect. The probability to die of a cell *i* that has accumulated k driver mutations, d^k^_i_ is given by:

$$d_{i}^{k}=\frac{1}{2}{\left(1-s\right)}^{k}$$

where s is the fitness parameter. According to the previous equation, the replication probability for the cell i with k mutations is b^k^_i_ = 1 − d^k^_i_. μ and s are the input parameters of the model and remain the same during the simulation and for all cells. The probability to die will change during the simulation depending on the number of accumulated driver alterations.

Given the available mutation data for human samples is limited to the exome, we estimated the mutation rate across multiple tumor types by assessing the number of mutations per nucleotide of the coding genes in the TCGA cohort. In our dataset, the number of mutations per nucleotide ranged between 7x10^-8^ to 10^−4^ (assuming an exome length equal to 6x10^7^, corresponding to ~2% of the genome length). Accordingly, we generated simulation with μ ∈ [10^−7^ − 10^−3^], which covers the estimated range in human tumors allowing for even higher mutation rate values. Similarly, variable fitness values have been previously proposed ranging between 0.0001 and 0.1 [43], [44]. In our simulations we reflected this variability setting *s* ∈ [10^−4^ − 5x10^-1^]. Finally, the probability for a new mutation to be a driver is defined as μ x K, with K = 0.025, chosen based on an estimation of 500 cancer associated alterations (e.g. as in COSMIC Cancer Census: http://cancer.sanger.ac.uk/census).

In our analyses, after each replication step, if no alteration has occurred then the two daughter cells will remain in the same clone, otherwise the sibling with the new alteration will create a new clone. Importantly, a new clone is formed whether the new alteration is a driver or a passenger. To calculate the mean number of clones and Tree score, only clones with a number of cells greater or equal to 1% of the total population are retained. This is in accordance with the fraction of sequencing reads typically required by cancer exome sequencing studies to retain a somatic mutation (S7 Fig). The model of clonal evolution is implemented in Python, using the ETE environment.

### Statistical tests

#### Enrichment analysis of mutational signatures and number of clonal and subclonal mutations

A score was derived for each patient and each mutational signature using the deconstructSig algorithm [37]. The score returned by this tool is proportional to the fraction of mutations that can be explained by the given signature normalized between 0 and 1. The tool was used with default parameters and using the exome2genome normalization as suggested by the authors (see https://github.com/raerose01/deconstructSigs). Next, we set all scores greater than 0 to 1, thus to obtained a binary matrix with signature calls for each patient: m[i,j] = 1, if patient i exhibit signature j, m[i,j] = 0, otherwise.

The association between signatures and the number of clonal and subclonal mutations observed in a tumor sample was tested by Wilcoxon one-tailed test: for each signature we tested whether tumors that exhibit the signature had a higher number of clonal (subclonal) mutations than tumors that did not. Each tumor type was tested separately and false discovery rate was controlled independently in each tumor type. Signatures were scored based on the -log10 of their p-value (Fig 4B), and signatures with a score > 2.5, which guaranteed a FDR < 0.1 in all tumor types, were retained as significant.

#### Enrichment analysis of selected alterations and number of clonal and subclonal mutations

For this analysis, we used a set of 505 cancer associated recurrent mutations and copy number alterations that we previously derived [38] and available at http://ciriellolab.org/select/select.html. This alteration set is formatted as a binary matrix such that: m[i,j] = 1, if patient i exhibit the alteration j, m[i,j] = 0, otherwise. Based on this binary matrix representation, we tested whether tumor exhibiting a given alterations had a higher number of clonal (subclonal) mutations using a Wilcoxon one-tailed test. Alterations were tested and scores using the same procedure we adopted for mutational signatures and retained as significant if their score > 3 (p-value < 0.001).

#### Survival analysis

Survival analysis was performed using the Python package *lifelines* (https://doi.org/10.5281/zenodo.1252342). P-values were computed by log-rank test.

## Supporting information

S1 Fig A) Tumor types and B) tumor subtypes ranked by the median of the mean reads per mutated site (mean RMS) of each sample (blue dots). The thick central line of each box plot represents the median number of significant motifs, the bounding box corresponds to the 25th–75th percentiles, and the whiskers extend up to 1.5 times the interquartile range.
(PDF)

S2 Fig For each tumor type, the number of altered copy number segments (X-axis) and the number of mutations (Y-axis) in each sample are compared by scatterplot. Samples are classified and color coded based on having more or less than 80 altered copy number segments (vertical blue line) and more or less than 300 mutations (horizontal blue line).
(PDF)

S3 Fig Number of clones estimated by EXPANDS in classes M, C, MC, and Low Instability. Samples are color coded by the their total number of mutations, with warm colors corresponding to high number of events. Samples with the highest number of mutations (red dots) have highest numbers of clones, consistent with the reported bias of EXPANDS for predicting high number of clones in tumors with high number of mutations.
(PDF)

S4 Fig **A)** Tree score as a function of the number of clones. From left to right, samples belonging to the MC (orange), M (green), and C (red) class are highlighted. The remaining samples are in the background (grey). **B)** Boxplot comparison of Tree scores in tumor samples with mutational instability (LUAD, SKCM, MSI, and POLE) and chromosomal instability (UCEC_CN High, BRCA Basal, STAD CIN). The thick central line of each box plot represents the median number of significant motifs, the bounding box corresponds to the 25th–75th percentiles, and the whiskers extend up to 1.5 times the interquartile range.
(PDF)

S5 Fig **A-D)** Kaplan-Meier curves comparing overall survival of patients from the pancreatic (A), liver (B), renal clear cell (C), and squamous-cell esophageal (D) cancer cohorts. Patients are stratified based on their Tree score being above (high, red curve) or below (low, black curve) the mean Tree score value of the corresponding tumor type. For each group, we report the corresponding mean number of clones and mean Tree score. Log-rank p-values are reported in bracket below the tumor type acronym. **E)** For each tumor type, we compared the percentage of surviving patients at the median time point (median follow-up of the cohort) for patients with high (>0.6) and low (<0.3) Tree scores. Each bar is the difference between these two values, positive values are in red (higher survival in high Tree score group), negative values in black (higher survival in low Tree score group). **F)** Boxplot comparison of R^2^ model fit value among tumor types. Samples with R^2^ model fit values above 0.98 (red line) are considered exhibiting features of neutral evolution.
(PDF)

S6 Fig Assessment of the accuracy of phylogenetic reconstruction using TCGA dataset. Scatter plots for the average number of reads per mutation and number of mutations per clone for: **A**) Inferred number of clones less than 3. **B**) Inferred number of clones equal to 3. **C**) Inferred number of clones equal to 4. **D**) Inferred number of clones equal to 5. **E**) Inferred number of clones equal to 6. **F**) Inferred number of clones greater than 6. The dashed lines represent the threshold line of exact subclonal reconstruction using synthetic data [24]. Samples above the threshold are correctly reconstructed. Points are color coded by density with low number of samples in blue and high number of samples in red.
(PDF)

S7 Fig Estimation of the detection threshold. Rank plot of the variant allele frequencies (VAF) of point mutation in TCGA dataset. No mutations are observed with a VAF lower than 1%.
(PDF)

S1 Table Properties of the tumor samples. Column 1—Tumor sample nameColumn 2—Tumor typeColumn 3—Tumor subtypeColumn 4—Mean number of reads per mutated siteColumn 5—Number of mutationsColumn 6—Number of copy number altered segmentsColumn 7—Top scoring phylogenies mean number of clonesColumn 8—Top scoring phylogenies mean Tree scoreColumn 9—Top scoring phylogenies mean number of clonal mutationsColumn 10—Top scoring phylogenies mean number of subclonal mutationsColumn 11—TCGA curated tumor sample purityColumn 12—ABSOLUTE inferred tumor sample purity.
(XLSX)

S2 Table Signature and alteration enrichment analysis for clonal and subclonal mutations. Tab 1: Signature enrichment results
Tab 2: Alteration enrichment results
Column names are consistent in both tabs:
Column 1—Signature ID (Tab 1) / Alteration ID (Tab 2)Column 2—Association with the number of clonal mutations (Wilcoxon p value)Column 3—Association with the number of subclonal mutations (Wilcoxon p value)Column 4—Tumor type where the test was madeColumn 5—Class: C, patient exhibiting the signature/alteration have significantly more clonal mutations, S, patient exhibiting the signature/alteration have significantly more subclonal mutations, CS, patient exhibiting the signature/alteration have significantly more clonal and subclonal mutations.
(XLSX)
