# Supplementary figures and images for: Pan-cancer inference of intra-tumor heterogeneity reveals associations with different forms of genomic instability
Source: PLoS Genet. 2018 Sep 13;14(9):e1007669. doi: 10.1371/journal.pgen.1007669 (PMC6155543; doi:10.1371/journal.pgen.1007669)

Supplementary Figure 1

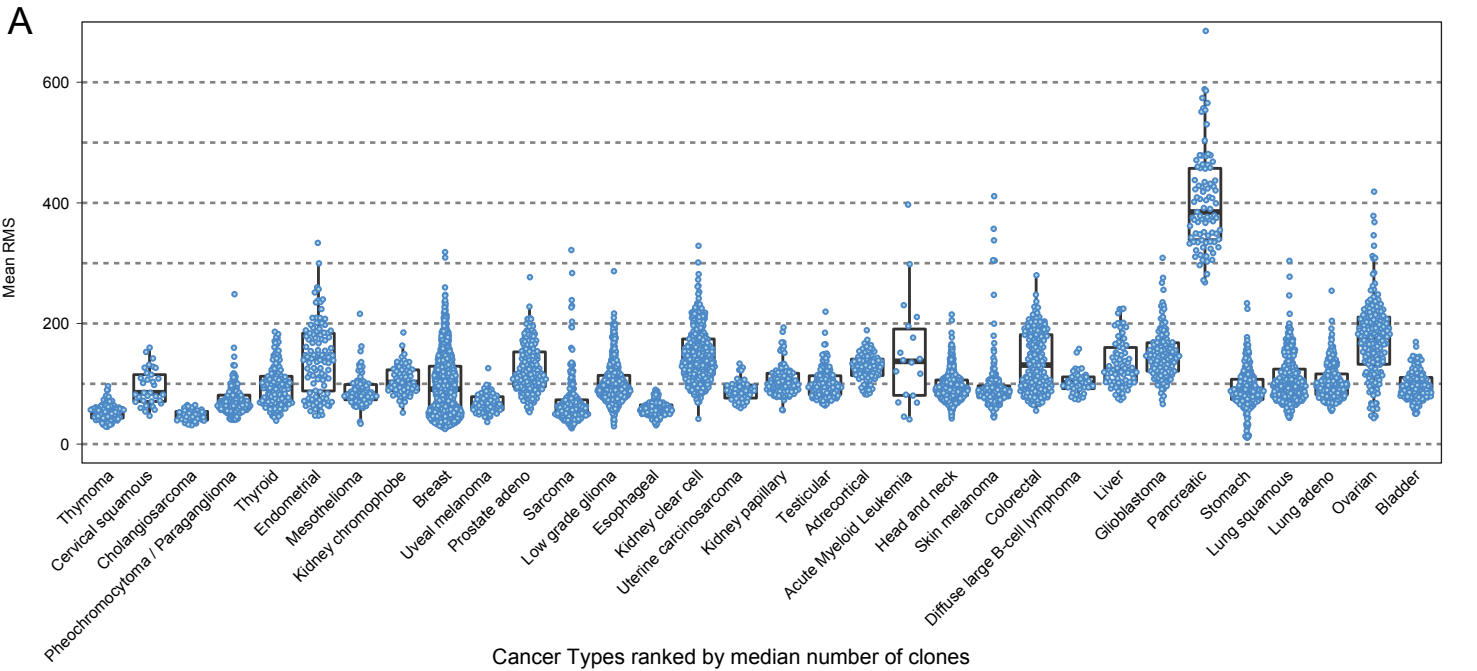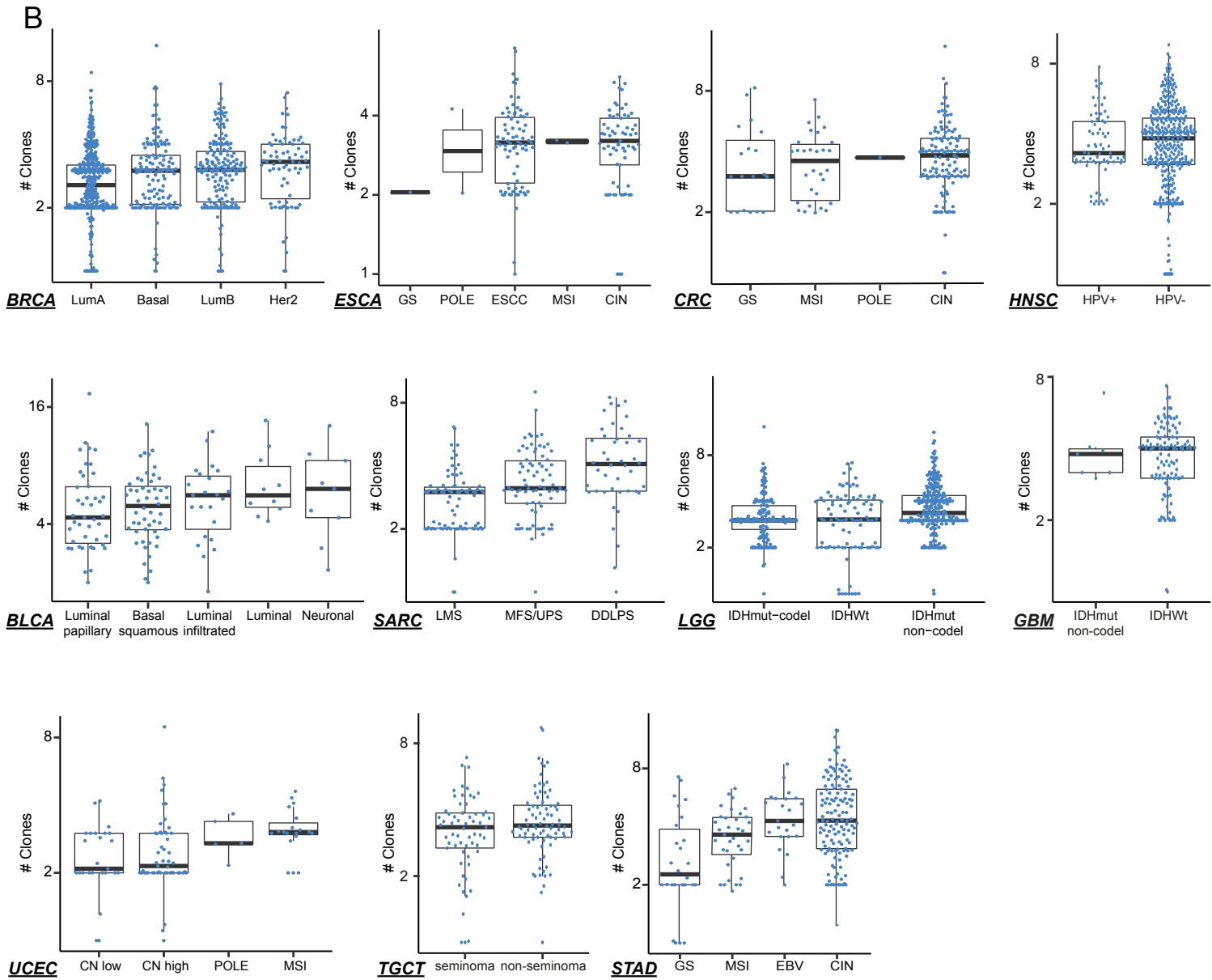

Supplement: S1 Fig — The thick central line of each box plot represents the median number of significant motifs, the bounding box corresponds to the 25th–75th percentiles, and the whiskers extend up to 1.5 times the interquartile range. (PDF) [file pgen.1007669.s001.pdf]

Supplementary Figure 2

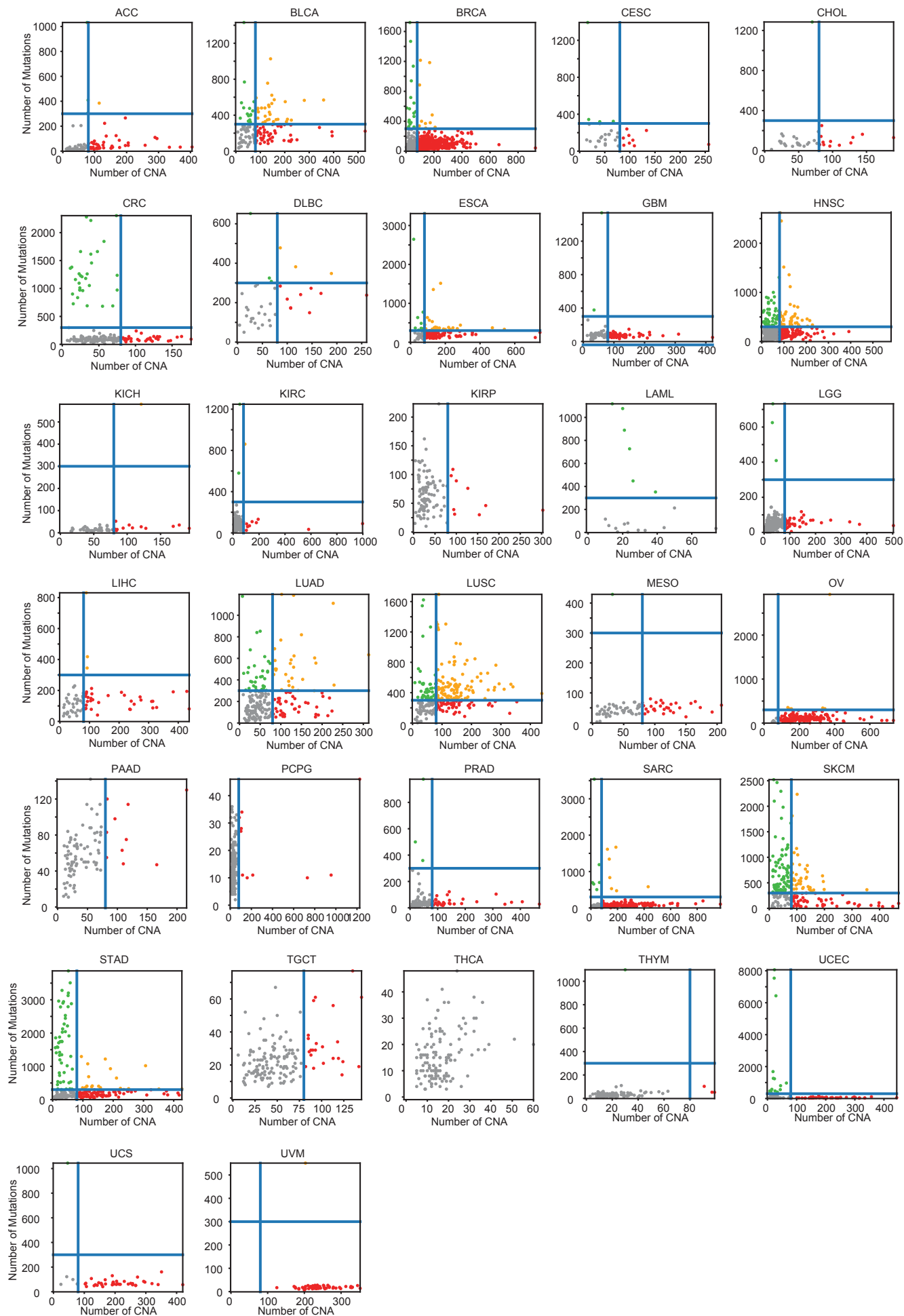

Supplement: S2 Fig — Samples are classified and color coded based on having more or less than 80 altered copy number segments (vertical blue line) and more or less than 300 mutations (horizontal blue line). (PDF) [file pgen.1007669.s002.pdf]

Supplementary Figure 3

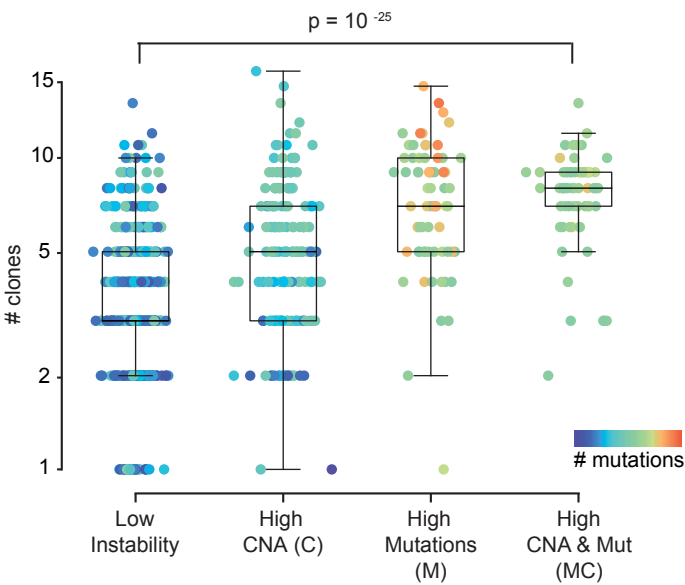

Supplement: S3 Fig — Samples are color coded by the their total number of mutations, with warm colors corresponding to high number of events. Samples with the highest number of mutations (red dots) have highest numbers of clones, consistent with the reported bias of EXPANDS for predicting high number of clones in tumors with high number of mutations. (PDF) [file pgen.1007669.s003.pdf]

Supplementary Figure 4

A

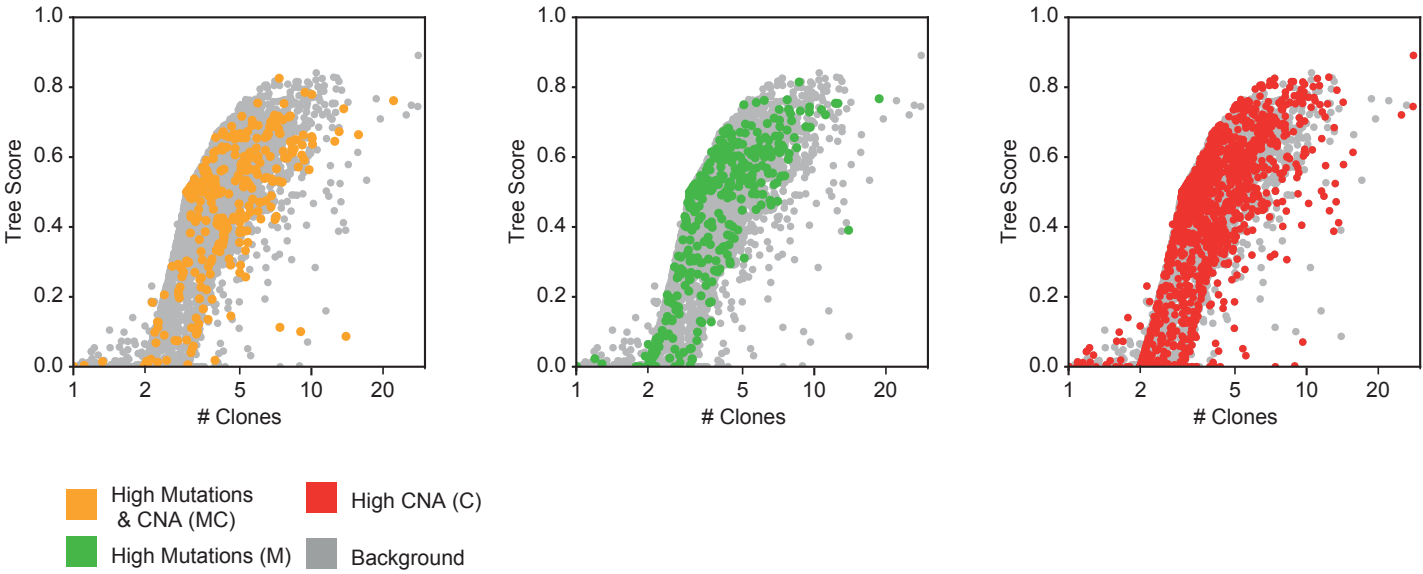

B

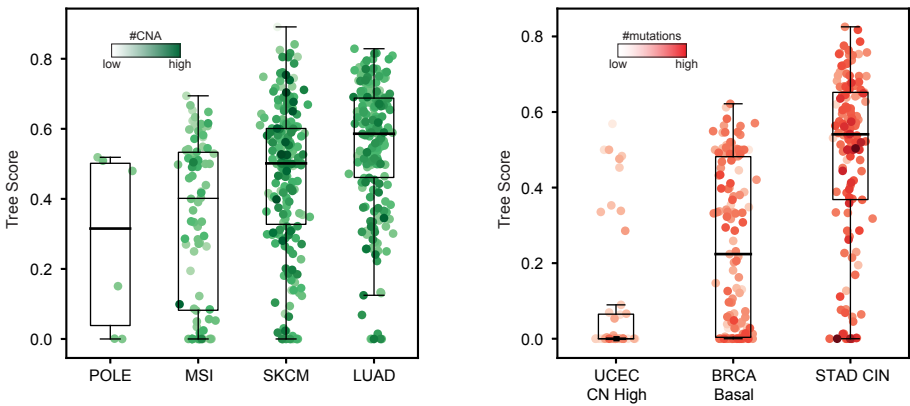

Supplement: S4 Fig — A) Tree score as a function of the number of clones. From left to right, samples belonging to the MC (orange), M (green), and C (red) class are highlighted. The remaining samples are in the background (grey). B) Boxplot comparison of Tree scores in tumor samples with mutational instability (LUAD, SKCM, MSI, and POLE) and chromosomal instability (UCEC_CN High, BRCA Basal, STAD CIN). The thick central line of each box plot represents the median number of significant motifs, the bounding box corresponds to the 25th–75th percentiles, and the whiskers extend up to 1.5 times the interquartile range. (PDF) [file pgen.1007669.s004.pdf]

A

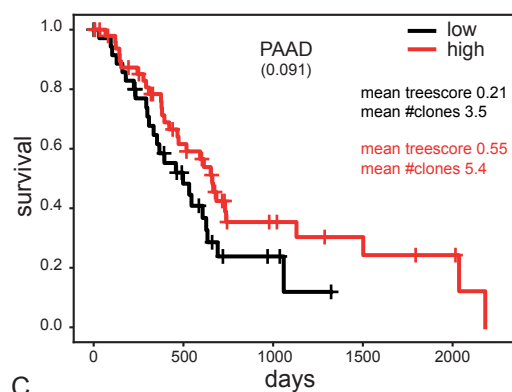

B

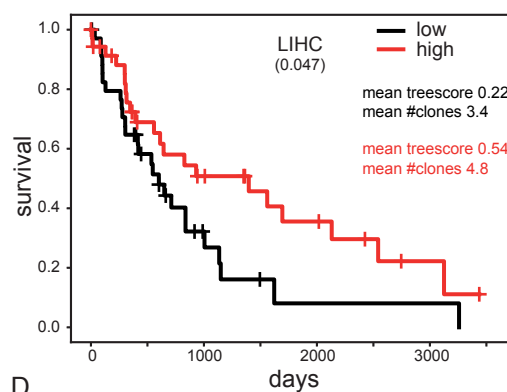

C

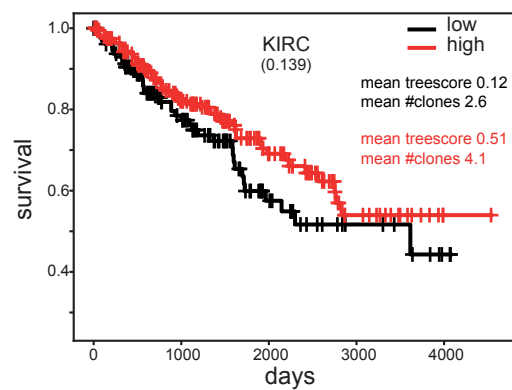

D

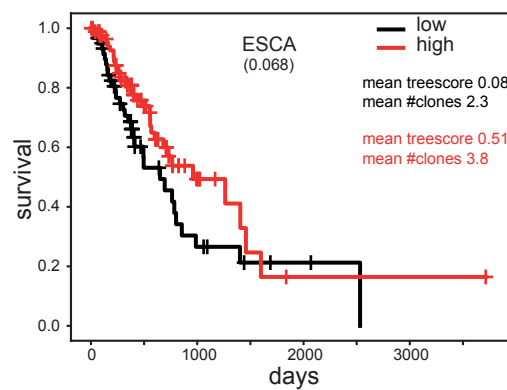

E

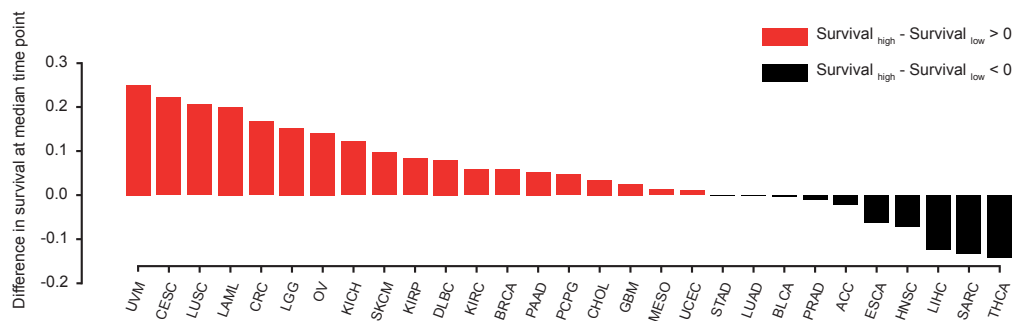

F

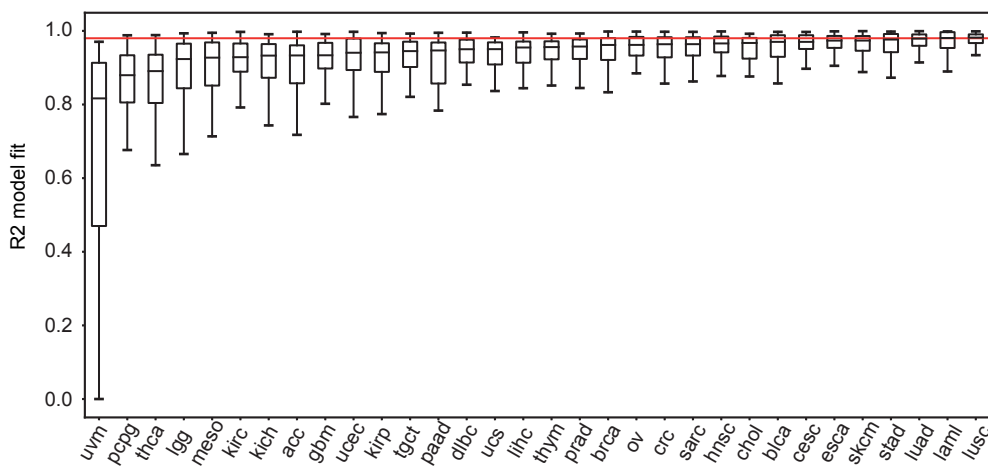

Supplement: S5 Fig — A-D) Kaplan-Meier curves comparing overall survival of patients from the pancreatic (A), liver (B), renal clear cell (C), and squamous-cell esophageal (D) cancer cohorts. Patients are stratified based on their Tree score being above (high, red curve) or below (low, black curve) the mean Tree score value of the corresponding tumor type. For each group, we report the corresponding mean number of clones and mean Tree score. Log-rank p-values are reported in bracket below the tumor type acronym. E) For each tumor type, we compared the percentage of surviving patients at the median time point (median follow-up of the cohort) for patients with high (>0.6) and low (<0.3) Tree scores. Each bar is the difference between these two values, positive values are in red (higher survival in high Tree score group), negative values in black (higher survival in low Tree score group). F) Boxplot comparison of R2 model fit value among tumor types. Samples with R2 model fit values above 0.98 (red line) are considered exhibiting features of neutral evolution. (PDF) [file pgen.1007669.s005.pdf]

Supplementary Figure 6

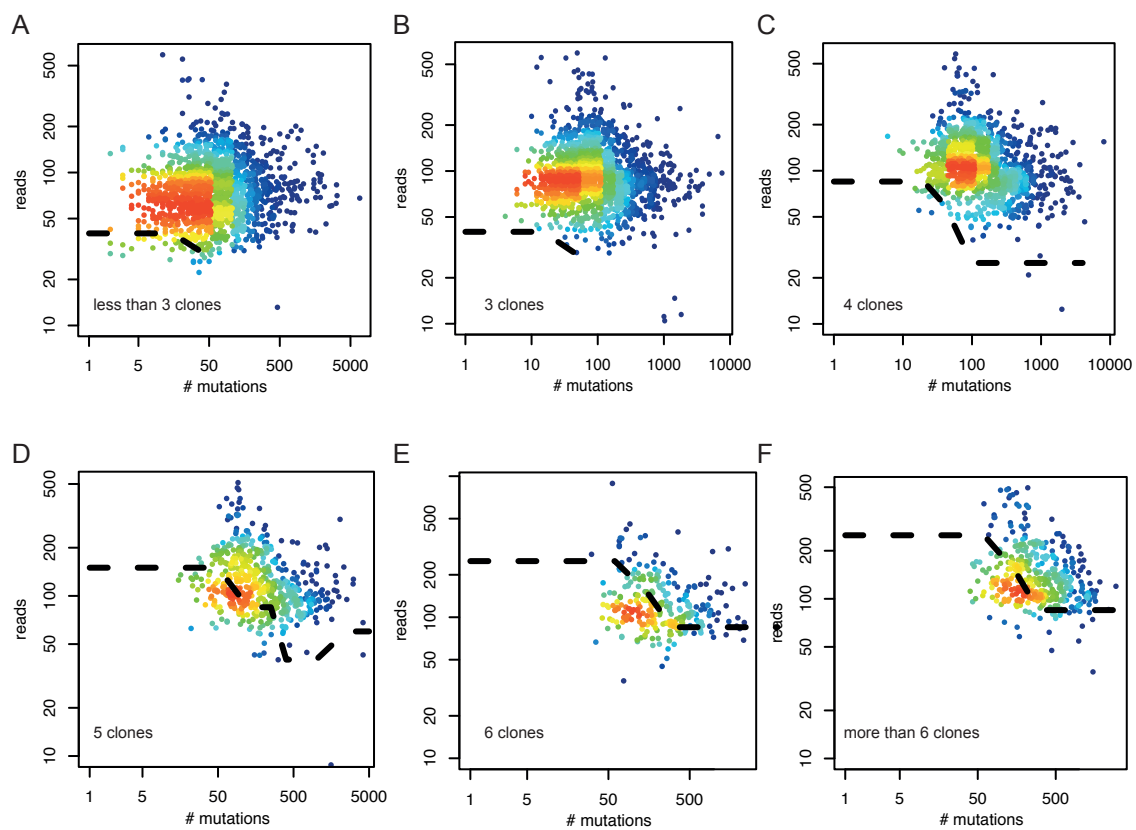

Supplement: S6 Fig — Scatter plots for the average number of reads per mutation and number of mutations per clone for: A) Inferred number of clones less than 3. B) Inferred number of clones equal to 3. C) Inferred number of clones equal to 4. D) Inferred number of clones equal to 5. E) Inferred number of clones equal to 6. F) Inferred number of clones greater than 6. The dashed lines represent the threshold line of exact subclonal reconstruction using synthetic data [24]. Samples above the threshold are correctly reconstructed. Points are color coded by density with low number of samples in blue and high number of samples in red. (PDF) [file pgen.1007669.s006.pdf]

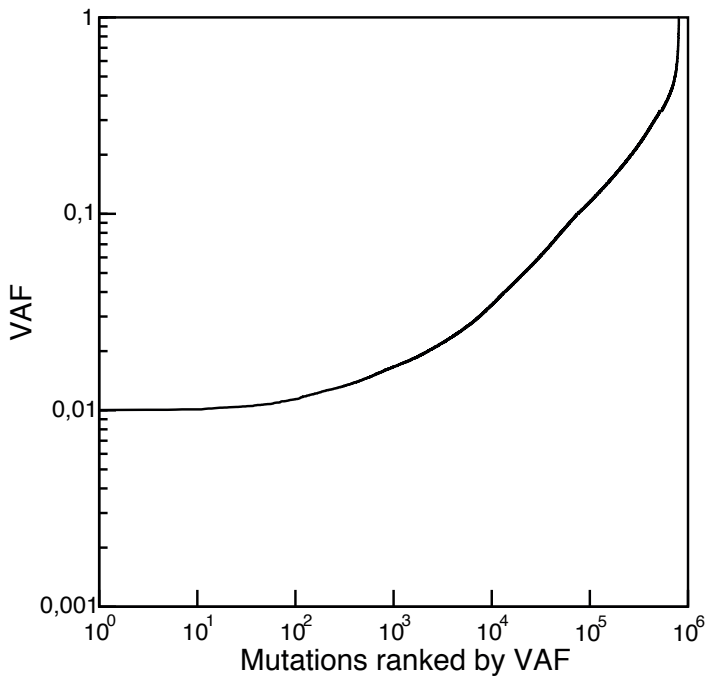

Supplement: S7 Fig — Rank plot of the variant allele frequencies (VAF) of point mutation in TCGA dataset. No mutations are observed with a VAF lower than 1%. (PDF) [file pgen.1007669.s007.pdf]
